# The Relationship Between Symptoms of ADHD, Mind Wandering, and Task Performance Among Kindergarten-Aged Children

**DOI:** 10.3390/bs15111439

**Published:** 2025-10-23

**Authors:** Yvette Pasternak Barami, Liat Goldfarb

**Affiliations:** Department of Learning Disabilities, University of Haifa, Mount Carmel, Haifa 31905, Israel

**Keywords:** mind-wandering, ADHD, kindergarten academic related performance

## Abstract

**Objective**: The association between Mind-Wandering (MW), symptoms of Attention-Deficit/Hyperactivity Disorder (ADHD), and task performance is understudied in children, and has never been studied in a population of kindergarten-aged children. Kindergarten is an important developmental stage in which children begin to acquire the building stones for proper academic abilities. **Methods**: One hundred and six kindergarten-aged children performed arithmetic and phonological tasks, and their level of MW was examined after each task in two different sessions. In addition, the ADHD symptoms’ level was collected for each participant. **Results**: A positive correlation between symptoms of ADHD and MW was found. In addition, reliability assessment of the two probes of MW indicated adequate reliability. Finally, the results suggest a connection between MW and academic-related task performance, beyond the effect of ADHD symptoms. **Conclusions**: MW is a stable cognitive structure beyond a specific task or time; it is also associated with symptoms of ADHD and relates to poorer performance in academic-related tasks in kindergarten-aged children.

## 1. Introduction

The human mental experience is fluid and may flow unintentionally between contents from both intrinsic and extrinsic sources. When the mind wanders due to an intrinsic source, the attention drifts unintentionally from its current train of thought to mental content generated by the individual (J. Smallwood & Schooler, 2015). The term that has been used to describe this this experience is self-generated thoughts (J. Smallwood, 2013a, 2013b; J. Smallwood & Schooler, 2015).

When a person performs a task, the attention system can shift from processing the task-related stimuli to internal information: such as daydreaming, memories, boredom, and worries (e.g., J. M. Smallwood et al., 2003). In this case, attention is “away from the current situation” (Giambra, 1995). A shift in the contents of thoughts away from an ongoing task or events in the external environment to self-generated thoughts and feelings can be termed Mind-Wandering (J. Smallwood & Schooler, 2015).

### 1.1. MW and Performance Impairment

MW is a common phenomenon; it has been suggested that in adults the mind wanders approximately 50% of one’s waking time (Killingsworth & Gilbert, 2010). Studies on MW suggest that it can impair performance in a variety of ways and relate to some impairment of performance in the task. For example, increased MW, both task related and task unrelated, was found to be related to an increase in RT variability (Stawarczyk et al., 2011) and disruption in reading and text comprehension (e.g., Frank et al., 2015). Similarly, it was found that MW can impair performance in a working memory capacity task, a fluid intelligence task, and in the Scholastic Aptitude Test (SAT; Mrazek et al., 2012), as well as in text comprehension (J. Smallwood et al., 2008), response inhibition (Kam & Handy, 2014), and sustained attention (Stawarczyk et al., 2011).

Neurologically, this impairment in task performance can be related to the default mode network (DMN). The DMN is a network of brain regions that includes the posterior/precuneus cingulate cortex and the medial pre-frontal cortex, found to be activated when one is not attending to an external task (Buckner et al., 2008). Interestingly, increased subjective reports on MW have found it to be strongly correlated with reduced deactivation of the DMN during on-task conditions (e.g., Mason et al., 2007; McKiernan et al., 2006).

In addition, the impairment in task performance that is associated with MW can be explained by several theories. For example, according to the executive-resource hypothesis (J. Smallwood & Schooler, 2006), task-related as well as task-unrelated MW share the same mechanism and compete for the same limited mental resources. Tasks that rely significantly on controlled processing leave fewer working memory resources available for MW, and vice versa.

### 1.2. The Connection Between MW and Symptoms of ADHD

Attention-Deficit/Hyperactivity Disorder (ADHD) is a neurodevelopmental disorder typically manifested early in the child’s development; however, formal diagnosis is often made around age 7, even though symptoms may emerge earlier (Miller et al., 2023). According to the Diagnostic and Statistical Manual of Mental Disorders (DSM-5), the disorder includes inattentive and hyperactive/impulsive symptoms (American Psychiatric Association, 2013), and it has been suggested that poor behavioral inhibition is a central deficiency in ADHD (e.g., Barkley, 1997). The prevalence of the disorder among children is approximately 5–7% (e.g., Polanczyk & Rohde, 2007), and it relates to academic impairments as well as other non-academic impairments such as social impairments (e.g., see Barkley et al., 2006; Loe & Feldman, 2007 for a review). Parental reports further suggest that inattention in ADHD is frequently attributed to underlying cognitive or motivational difficulties (Keulers & Hurks, 2021). Recently, a variety of studies have suggested that MW is a significant independent characteristic of ADHD.

Recent studies have also found that MW in general relates to ADHD. Alali-Morlevy and Goldfarb (2021) found that both task-related and task-unrelated MW interfere with performance on a sustained attention task. University students with ADHD had a greater tendency to MW, compared to participants with no diagnosis of ADHD. In another study that examined the levels of MW after performing a selective attention task, university students with diagnosed ADHD were found to have increased levels of MW (Alali-Morlevy & Goldfarb, 2022).

Participants were found to report more MW when they had more symptoms of attention deficiency, even in cases of participants with no clinical diagnosis of ADHD but still showing some symptoms. For example, Franklin et al. (2017) found that in a group of adult students with typical development, those who reported more ADHD symptoms also reported more MW: on a computerized attention task, in a reading task, and in general daily life tasks. Similar results were found in other adult student populations (e.g., Arabacı & Parris, 2018; Seli et al., 2015; Shaw & Giambra, 1993).

### 1.3. The Current Study

The current study focuses on kindergarten-aged children. Kindergarten is the stage before formal schooling begins and it is when children establish the foundations of proper academic abilities such as reading and numeracy and display a range of proficiency levels (e.g., Duncan et al., 2007; Kendeou et al., 2009).

Different cognitive structures and abilities that promote proper learning, such as self-direction, self-regulation, persistence, motivation, and attention, have been studied in young children (e.g., Claessens et al., 2009; Foulks & Morrow, 1989; Lin et al., 2003; McClelland et al., 2006; Shonkoff et al., 2000). However, MW and its connection to other cognitive structures have not been thoroughly examined in kindergarten-aged children samples. Recently, emerging work with slightly older children has begun to address this gap. For example, Cherry et al. (2022) found that MW in children aged 6–11 predicted poorer memory retention, and that higher interest in the material reduced MW and improved recall. In addition, Cherry et al. (2024) linked MW in school-aged children to both academic motivation and executive functions. Wiggs et al. (2024) found that teacher-rated cognitive disengagement, but not ADHD-inattentive symptoms, predicted probe-caught MW in children. However, the study did not assess hyperactive-impulsive symptoms and focused on children in grades 4–6. Together, these studies begin to situate MW within learning contexts in children, though research specifically focused on kindergarten remains scarce. Beyond its relation to learning and motivation, another critical line of research has examined the potential links between MW and symptoms of ADHD.

As noted above, while a variety of studies on adults have found a connection between MW and symptoms of ADHD (for e.g., Alali-Morlevy & Goldfarb, 2021, 2022; Arabacı & Parris, 2018; Franklin et al., 2017; Kooij et al., 2019; Mowlem et al., 2019; Seli et al., 2015), as well as deficiencies in task performance as a result of MW (e.g., Kam & Handy, 2014; Mrazek et al., 2012; J. Smallwood et al., 2008; Stawarczyk et al., 2011), this association is understudied in children and the results are mixed. The first study, which explored this relationship among children with a mean age of 9 (SD = 1.82), investigated MW during task performance in four groups: children with ADHD who had never received treatment with stimulant drugs, children with ADHD who were medicated with stimulant drugs, a control group with typical development, and another control group with psychiatric conditions other than ADHD. They found increased MW only among the ADHD group medicated with stimulant drugs (Van den Driessche et al., 2017). The second study, which explored this relationship among children with an average age of 10.46 (SD = 1.35), found that MW was associated with higher levels of ADHD symptoms in children diagnosed with ADHD (Frick et al., 2020).

ADHD-related attentional deficits involve general attentional lapses that can result from various causes, such as external distractors, but are not limited to them. Specifically, MW refers to attentional lapses that relate to internally generated thoughts that drift away from the task at hand (J. Smallwood & Schooler, 2015). Recent studies suggest MW is better regarded as a distinct construct: it is highly prevalent among adults with ADHD (Mowlem et al., 2019), may predict ADHD more strongly than core symptoms (Kooij et al., 2019), and has been argued to represent an independent and significant characteristic of ADHD (Alali-Morlevy & Goldfarb, 2021, 2022). Van den Driessche et al. (2017) were the first to study MW in children, defining it as spontaneous, task-unrelated thoughts with rich content and independence from environmental distractions. The present study focuses specifically on such inner task-unrelated thoughts, as they best capture the essence of MW and its potential association with ADHD symptomatology.

Regarding the connection between deficient task performance and excessive MW, this line of study is understudied in children too. Mrazek et al. (2013) found in a group of young children ages 11–13 that increased MW during testing was correlated with poorer reading comprehension performance as well as poorer well-being. Soemer et al. (2019) also found a correlation between increased MW and poorer text comprehension in eighth grade students with a mean age of 13.91 (SD = 0.72). Another study conducted on children with neurotypical development with a mean age of 10.12 (SD = 0.42) found that children with poorer inhibition performance were prone to more MW (Keulers & Jonkman, 2019).

Note that all previous studies did not examine these important questions in young children such as kindergarten-aged children. The current study is the first to examine MW at this important age. Specifically, the current study examined the association between MW and symptoms of ADHD, as well as between impairment in academic-related task performance and MW, in kindergarten-aged children.

In the current study, MW was examined in each child after performing two tasks: an arithmetic task and a phonological task, in two different sessions. The performance level in these tasks was also examined. Note that the current study did not examine a population diagnosed with ADHD, but a trait symptomology of ADHD in everyday life, which was assessed for each child as a continuum that described the severity of the trait.

Previously it has been suggested that ADHD-related symptoms can be characterized as a continuum trait (e.g., Franklin et al., 2017; Overbey et al., 2011; Whalen et al., 2003). In addition, the number of ADHD-related symptoms was also found to be correlated with academic achievement among children, whose scores were below the clinical threshold for diagnosis (e.g., Currie & Stabile, 2006). It is important to note that children with symptoms of ADHD and with no formal diagnosis can also suffer significant negative consequences due to ADHD symptomology (Currie & Stabile, 2006; Loe & Feldman, 2007). This understanding is critical, considering that there are still many underdiagnosed people (Kooij et al., 2019; Rucklidge, 2010). Recent community-based work with preschool children further highlights this point, showing that ADHD prevalence in kindergarten-aged populations ranges from 2% to over 12%, depending on the assessment method, with clinical diagnoses at around 3% (Ozturk et al., 2025). This increases the importance of all efforts at understanding children with milder symptoms. Therefore, the current work refers to ADHD symptoms as a continuum trait symptomology of ADHD.

In light of the above, the current study examines the association between symptoms of ADHD and MW among kindergarten children. If the relationship between symptoms of ADHD and MW among older participants is also characteristic of kindergarten children, then it is hypothesized that elaborate MW will be associated with more symptoms of ADHD. The second purpose is to examine the association between MW and academic-related task performance in kindergarten children. This association is examined too—beyond the potential effect of symptoms of ADHD on this relationship. If an increase in MW is associated with a decrease in task performance beyond the effect of increased levels of ADHD symptoms, a significant direct effect is expected for the MW variable.

## 2. Method

### 2.1. Participants

One hundred and six participants, with a mean age of 5 and 8 months (SD = 0.39, 53.7% females) were recruited from general-education kindergartens in the greater Haifa area (northern Israel); special-education settings were excluded. The sample size of the present study was based on a previous large community sample that examine similar questions in adults (i.e., Franklin et al., 2017). The study was approved by the Office of the Chief Scientist in Israel.

Participants were recruited as part of a larger data collection effort at the Edmond J. Safra Research Center for the Study of Learning Disabilities. This broader project included a range of academic, emotional, and cognitive assessments administered by different researchers to study different aspects of children’s academic developments. At the beginning of the experiment, the parents confirmed their child’s participation by signing a consent form. The responses of ten participants were not included in the study either because they did not complete the two interfering thoughts questionnaires (seven participants) or because they answered “no” to all the questions (three participants), which raised a suspicion that they were not engaged with the questions. All participants were native Hebrew speakers. None of the children had a formal ADHD diagnosis at that point in time, since diagnoses for ADHD are more commonly given at a later stage in life, after starting school.

### 2.2. Assessment

In order to investigate symptoms of ADHD, the parents of the participants were asked to complete a questionnaire based on the Diagnostic and Statistical Manual of Mental Disorders–DSM-5 Attention-Deficit/Hyperactivity Disorder (ADHD) Criteria Questionnaire (American Psychiatric Association, 2013). The questionnaire consisted of 18 items, divided into inattention and hyperactivity/impulsivity domains. Parents indicated the presence or absence of each symptom (yes/no) based on their child’s behavior. Each affirmative response was scored as one point, allowing for the calculation of symptom counts per child. Importantly, this checklist was designed to identify ADHD-related traits rather than to provide a severity index, which was consistent with the goals of the present study, and is commonly used in research to examine trait symptomatology of attention in everyday life. In order to investigate MW, the thinking content component of the Dundee Stress State Questionnaire was used (DSSQ; Maillet & Rajah, 2013; Matthews et al., 1999; J. Smallwood et al., 2005, 2004b). This questionnaire had previously been translated into Hebrew and used to assess MW (e.g., Alali-Morlevy & Goldfarb, 2021). The questionnaire consists of 14 statements of MW and includes different subjective experiences of MW that is related and unrelated to the task, such as “I thought about how my friends did on this task” or “I thought about members of my family”. Self-generated thoughts can be task related and task unrelated (J. Smallwood & Schooler, 2015), and MW as a term mostly refers to both cases (but see Stawarczyk et al., 2011).

In the current study, the subjects completed the questionnaire twice; once after the arithmetic task and once more after the phonological task (4 months apart on average). In the original version of this questionnaire the response scale was from 1–5; however, the current study was conducted with young children. It has been suggested that the most effective way of presenting questionnaires to children is with simple sentences and items consisting of a three-response format (Royeen, 1985). In addition, a pilot experiment that we conducted prior to this study on kindergarten children, confirmed that a 1–5 scale is too confusing for young children. Hence, in the current study the response scale was modified to a scale of 1–3 (1—not at all, 2—only a little, 3—a lot)

In order to investigate task performance in a task in which the MW occurs, two tasks were used. The first was an arithmetic task (Shaul, 2017, based on Purpura et al., 2015) in which participants were asked to solve simple arithmetic problems, including addition and subtraction, where the results of the arithmetic problems did not exceed the number 8 (i.e., 2 + 1, 1 + 3, 2 + 2, 3 + 2, 7 + 1, 6 + 2, 4 + 3, 2 − 1, 4 − 2, 5 − 4). The experimenter read aloud the arithmetic problem and the participant gave a response. If the participant made three consecutive mistakes, the task was ended.

The second task was a phonological task (Nevet-Reznik et al., 2018). In this task the experimenter read a word aloud and the participant had to state the opening sound of the word. The previous literature has shown that kindergarten-aged children can categorize words on the basis of single phonemes, especially opening sounds that begin with a consonant (Kirtley et al., 1989; Treiman, 1985). In this study, for example, in the word “Little”—“ktzat” in Hebrew, the opening sound is “k”. Before beginning the task, participants underwent a practice task of two or three words to make sure that they understood the task. After five consecutive mistakes, the task was ended by the experimenter. The task contained 10 words, and the number of correct answers for each participant was calculated.

## 3. Results

For each participant, a score of MW was calculated by the sum of questions in which the participant reported experiencing MW at a high frequency (i.e., they scored 3). These values ranged from 0 to 14. This was calculated for each task and then averaged across the two tasks.

In addition, an attention deficiency score was calculated from a questionnaire based on the DSM-5 ADHD criteria (American Psychiatric Association, 2013). The questionnaire contained 18 statements, including both inattentive and hyperactive-impulsive characteristics. This questionnaire was completed by the participating children’s parents, who noted whether the statements were true or false regarding their child. Hence, the attention deficiency variable included values ranging on a continuum from 0–18.

A score was calculated for academic-related task performance as the mean of the correct answers on the arithmetic and phonological task together. Both tasks contained 10 items each; therefore, the values of this variable ranged from 0–10.

Since this is the first time that a MW questionnaire has been utilized with participants of a young age, such as kindergarten children, the reliability of MW was tested in the two sessions. Each session was devoted to a different task; one arithmetic and the other phonological, with an interval of 4 months on average. A Pearson correlation was computed to assess the reliability of the MW questionnaire, *r*(96) = 0.718. This indicates adequate reliability and strengthens the assumption of a relatively stable cognitive structure of MW in kindergarten-aged children, beyond the specific task type and beyond the time it was tested.

### 3.1. The Relationship Between Symptoms of ADHD and MW

**Figure 1:**
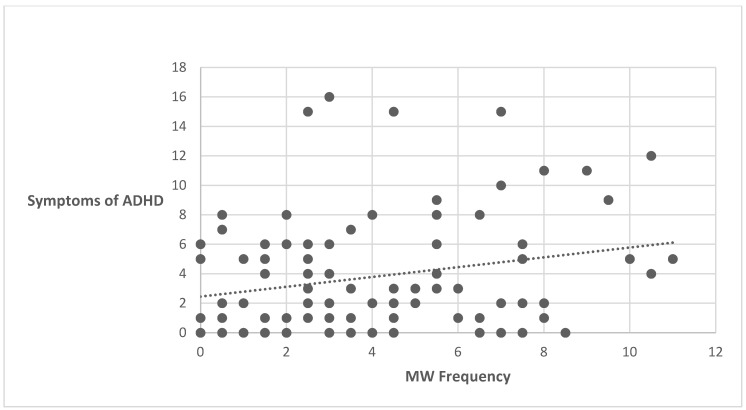
Correlation between symptoms of ADHD and the frequency of MW. Each dot represents one participant’s data point.

To examine whether there is a relationship between symptoms of ADHD and the frequency of MW, Pearson correlations were conducted between the variables. A significant positive correlation was found between overall ADHD symptoms and MW, *r*(94) = 0.239, *p* = 0.01, indicating that the higher levels of ADHD symptoms were associated with more frequent MW episodes. Each dot in Figure 1 represents one participant’s data point (see Figure 1).

To further examine whether this association differs between symptom domains, correlations were calculated separately for inattention (ADHD-I) and hyperactivity/impulsivity (ADHD-HI). Results indicated a significant positive correlation between ADHD-I symptoms and MW, *r*(94) = 0.18, *p* = 0.042, as well as ADHD-HI symptoms *r*(94) = 0.24, *p* = 0.0095. These two correlations did not differ significantly from each other, z = 0.63, *p* = 0.26.

### 3.2. The Association Between MW and Academic-Related Task Performance

**Figure 2:**
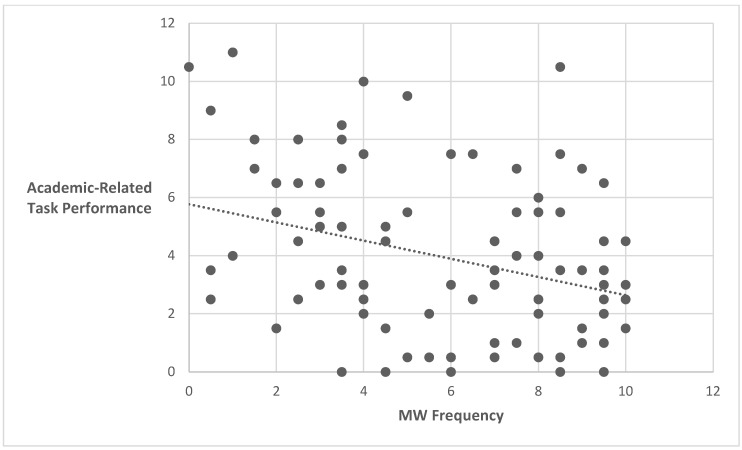
Correlation between academic-related task performance and MW frequency. Each dot represents one participant’s data point.

To examine whether there is a relationship between the frequency of MW and academic-related task performance, a Pearson correlation was conducted between the variables. A significant negative correlation was found between the variables, *r*(94) = −0.323, *p* = 0.001. Hence, the more MW was described in children, the lower the performance in academic-related tasks. Each dot in Figure 2 represents one participant’s data point (see Figure 2).

In order to examine whether there is an association between MW and performance in academic-related tasks beyond the contribution of symptoms of ADHD, a multiple regression analysis was performed.

Accordingly, a regression analysis with MW and symptoms of ADHD as predictors and academic-related task performance as the outcome variable found a significant goodness of fit, R^2^ = 0.109, F(2,93) = 5.707, MSE = 7.655, *p* < 0.01. MW had a significant and unique contribution to the explanation of the variability in the change in task performance (Beta = −0.306, *p* < 0.01), while symptoms of ADHD did not (Beta = −0.072, *p* = 0.475). Accordingly, the higher the frequency of MW, the lower the performance in the tasks, beyond the potential effect of symptoms of ADHD on this relationship.

## 4. Discussion

The current study examined the association between MW and symptoms of ADHD, as well as between impairments in academic-related task performance and MW, in children attending kindergarten. The results suggest that MW is associated with symptoms of ADHD in kindergarten-aged children. The more symptoms of ADHD were described, the more MW was found. In addition, a higher frequency of MW was related to lower performance in academic-related tasks. This association was examined beyond the potential effect of ADHD symptoms and a significant direct effect was found for MW on task performance.

Regarding the first finding: MW is associated with symptoms of ADHD; this study is the first to find this association in kindergarten-aged children. Previous studies with adults (Alali-Morlevy & Goldfarb, 2021, 2022; Arabacı & Parris, 2018; Franklin et al., 2017; Kooij et al., 2019; Mowlem et al., 2019; Seli et al., 2015) have indicated that MW is associated with symptoms of ADHD. As mentioned, Van den Driessche et al. (2017) were the first to explore this relationship in children. They found in a group of children aged 6–12 that increased MW was observed in the group diagnosed with ADHD that received medical treatment for more than a month. Frick et al. (2020) also found that MW was associated with higher levels of ADHD symptoms in children aged 8–13 diagnosed with ADHD.

The current study extends these findings, as it found this relationship at kindergarten age. This finding provides further evidence that more symptoms of ADHD may be accompanied by a bigger likelihood of MW. Therefore, some children may have an increased risk of MW at a very young age.

Furthermore, note that the current study found a reliability between the two sessions of the MW questionnaire. Each session was performed with a different task, one arithmetic and the other phonological, with an interval of 4 months on average. This strengthens the assumption of a relatively stable cognitive structure of MW beyond the specific task type or beyond the time it was tested, even in kindergarten-aged children. This is also consistent with previous studies that have shown that older children have the ability to introspect on their own thoughts with reasonable reliability (Frick et al., 2020; Mrazek et al., 2013; Van den Driessche et al., 2017).

Building on this point, the second aim of this study was to examine the connection between MW and academic-related task performance in kindergarten-aged children. Previous studies found that MW can impair a variety of measures related to task performance in adults. (e.g., Frank et al., 2015; Kam & Handy, 2014; Mrazek et al., 2012; J. Smallwood et al., 2005, 2008; Stawarczyk et al., 2011). In a sample of children aged 11–13, for example, it was found that increased MW was correlated with poorer reading comprehension and well-being during testing (Mrazek et al., 2013). Likewise, increased MW correlated with poorer text comprehension in eighth grade students (Soemer et al., 2019). MW was also found to be correlated with poorer inhibition performance in tasks, in a sample of children with a mean age of 10 (Keulers & Jonkman, 2019). The current study extends these findings again—to a much younger age—and targets performance on early literacy and numerical academic tasks. Complementary evidence from younger school-aged samples further highlights these links: Cherry et al. (2022) showed that MW frequency mediated the relation between children’s topic interest and memory performance—greater interest predicted less MW, which in turn predicted better recall. Extending this work, Cherry et al. (2024) demonstrated that higher MW was strongly associated with both immediate and one-week delayed recall, providing the first evidence of MW’s impact on delayed memory in children.

Kindergarten is a stage at which children begin to acquire the building stones of academic abilities such as phonological awareness and arithmetic. Early reading-related skills have an effect on later reading abilities, even after several years of formal instruction in elementary school (Hecht et al., 2000; Nicholson, 1997). Similarly, early arithmetic skills can affect later mathematical development (Hannula et al., 2010; Hannula-Sormunen, 2015). In addition, phonological awareness has been found to predict early arithmetic attainment (Simmons et al., 2008). Literacy abilities and math development are correlated from the early kindergarten years onward (Krajewski & Schneider, 2009; Simmons et al., 2008). Krajewski and Schneider (2009) found that the impact of phonological awareness assessed at 5 years of age predicted math achievement in third grade. Simmons et al. (2008) also assessed phonological awareness in children at 5 years of age and 12 months later. They found that phonological awareness was one of the significant independent predictors of arithmetic skills. Thus, these two academic abilities, phonological awareness and arithmetic, are related; therefore, the kindergarten years have great importance for understanding and advancing future academic functioning. Hence, identifying MW as a factor that is correlated with poorer task performance in early years might have an important potential contribution to later academic performance.

In addition, the current study also found a significant direct effect for MW on task performance, beyond the potential effect of symptoms of ADHD, identifying it as an important factor in predicting academic task performance. Frick et al. (2020) hypothesized that MW would contribute independently to academic functioning beyond core symptoms of ADHD as well as other factors; however, it failed to reach significance in that specific study. Frick et al. (2020) examined MW in general: in everyday life as well as general academic performance based on grade and teacher assessments. In contrast, the current study examined specific task performance and the MW that appeared in those specific tasks. This technique might have been more helpful in finding a stronger connection between these components. However, since the academic performance was measured by two tasks only, the ability to generalize the results to general academic performance is somewhat limited. Future studies could add additional indicators for the assessment of academic abilities, such as teacher evaluations, grades, and additional tasks related to reading and arithmetic skills. The additional tasks, however, may cause even more MW due to longer time performance.

Another possible methodological limitation that should be addressed is that MW was measured by a self-report questionnaire at the end of the experiment only. This is a common method in the literature on MW (Alali-Morlevy & Goldfarb, 2021), but the participant may forget the MW instances by the end of the task (Alali-Morlevy & Goldfarb, 2023). Another method for examining MW utilized in the research literature is real time online questionnaires, in which the participants are asked several times about their thoughts during the task, with constant breaks in the task (e.g., Bastian & Sackur, 2013; J. Smallwood et al., 2004a). This method makes participants aware of the nature of the investigation (e.g., Schooler et al., 2011; Seli et al., 2015). The awareness to MW combined with social priming (e.g., Goldfarb et al., 2011) may lead to exaggerated MW, especially in young children, since they may alter their behavior without awareness (Wilson et al., 2022). Therefore, special caution is needed for avoiding priming any particular response. Future studies could examine the contribution of additional variables in mediating the relationship between MW and academic performance among kindergarten children. For example, one limitation of the present study is the absence of an intelligence measure. All participants were enrolled in regular kindergartens and did not attend special-education programs; nevertheless, it is possible that the IQ factor could affect this connection. Similarly, other factors such as different executive functions (Wilson et al., 2022; Keulers & Jonkman, 2019), may further contribute to our understanding of the phenomenon.

In addition, it is also important to acknowledge the limitations associated with children’s metacognitive awareness. For instance, Cherry et al. (2022) noted that younger children may not always introspect reliably or may be hesitant to acknowledge deliberate engagement in MW. Young children may have limited capacity to accurately evaluate and describe their own internal cognitive experiences in hindsight, which could impact their ability to answer retrospective self-report questionnaires. Hence, complementary approaches such as probe-caught measures could therefore strengthen assessment in future studies. Additionally, investigating the development of metacognitive awareness in relation to mind wandering may provide valuable additional insights.

Finally, the findings of the current study might have possible future implications. Further research can be directed at studying questionnaires on MW as preliminary tools for mapping children with a potential tendency to mind wander as well as academic performance impairments. Nowadays, when there are deficiencies in children’s academic performance it is customary to examine the child’s intellectual abilities, the existence of learning disabilities, and/or the presence of ADHD. Yet, the current study suggests that measuring MW is also an important course of investigation. Additional measurements for predicting academic difficulties that are independent of ADHD diagnoses are needed as predictive markers to facilitate early identification and to support developmental interventions that promote health and well-being (Miller et al., 2023). In this case, questionnaires on MW may reliably be used for understanding task impairment performance through MW.

## Data Availability

We report on how we determined all data exclusions and on all measures used in the study. The datasets generated and analyzed during the current study are not publicly available due to privacy issues but are available from the corresponding author on reasonable request.
